# High Prevalence of Vitamin D Deficiency in Patients Undergoing Total Shoulder or Elbow Arthroplasty

**DOI:** 10.3390/nu17162635

**Published:** 2025-08-14

**Authors:** Miledi Hoxha, Tizian Heinz, Maximilian Rudert, Kilian List, Leonard Achenbach, Gerrit Maier, Manuel Weißenberger, Konstantin Horas

**Affiliations:** 1 Orthopaedic Center for Musculoskeletal Research, University of Wuerzburg, 97074 Wuerzburg, Germany; 2 Department of Orthopaedic Surgery, Koenig-Ludwig-Haus, University of Wuerzburg, 97074 Wuerzburg, Germany; t-heinz.klh@uni-wuerzburg.de (T.H.);; 3 Department of Orthopaedic Surgery, Pius-Hospital, Carl-von-Ossietzky-University, 26129 Oldenburg, Germany; 4 Orthopaedic Surgery Centre (OCW), 97070 Wuerzburg, Germany; 5 Frankfurt Centre for Bone Health and Endocrinology, 60313 Frankfurt, Germany

**Keywords:** vitamin D, vitamin D deficiency, total shoulder arthroplasty, joint replacement

## Abstract

Background: Vitamin D deficiency represents a global health problem of enormous extent. It is estimated that around one billion people worldwide have inadequate vitamin D levels. This phenomenon is directly associated with negative impact on a variety of orthopaedic conditions. Further, there is now robust evidence that perioperative vitamin D levels in patients scheduled for total joint replacement (TJA) affect outcome and the healing process. To date, only few studies focus on vitamin D levels of patients scheduled for total arthroplasty of the upper extremity (shoulder and elbow). For this reason, the objective of this study is to determine the prevalence of vitamin D deficiency in this patient collective. Methods: In a monocentric cohort study, serum levels of 25-hydroxyvitamin D (25OHD) were measured preoperatively in all patients undergoing total shoulder or elbow arthroplasty. Demographic and perioperative data as well as comorbidities were recorded from medical records to assess for potential risk factors for hypovitaminosis D. Multivariate regression analyses were used to identify risk factors for vitamin D insufficiency and deficiency. Results: Collectively, 108 patients with total joint replacement of the upper extremity were included over a period of twelve months. Notably, 28.7% (31/108) of patients reported a regular intake of vitamin D supplements. 62.3% (19/31) of those had sufficient vitamin D levels, while 38.7% (12/31) had insufficient and further 6% (2/31) deficient vitamin D levels (<20 ng/mL). Remarkably, 87% of patients that did not report a regular vitamin D intake (*n* = 77) showed low serum vitamin D levels. In particular, 63.6% (49/77) were vitamin D deficient, 23.4% (18/77) vitamin D insufficient and only 13% of patients had vitamin D serum levels above or equal to 30 ng/mL that are considered sufficient (mean serum 25(OH)D = 36.4 ng/mL for vitamin D substitution vs. 18.4 ng/mL for no substitution; *p* < 0.0001). Moreover, vitamin D levels varied between seasons, with the lowest levels recorded in spring (OR = 4.32, *p* = 0.044) and the highest levels in summer (*p* = 0.005 vs. spring). Conclusion: Patients undergoing total shoulder or elbow arthroplasty have an increased risk profile for hypovitaminosis D (vitamin D supplementation had 94% lower odds of being deficient; OR = 0.06, *p* = 0.001). Seasonal circumstances at the point of arthroplasty seem to be a key risk factor for low vitamin D levels. For this reason, it would be advisable to consider preoperative serum vitamin D level measurement as an integral part of the regularly performed preoperative care.

## 1. Introduction

Total shoulder arthroplasty (TSA) represents an effective treatment for severe shoulder diseases, including osteoarthritis, proximal humerus fractures, inflammatory joint conditions (e.g., rheumatoid arthritis), avascular necrosis, and rotator cuff arthropathy [1,2,3,4]. Incidences of performed shoulder replacements are expected to increase rapidly in the following years [2,3,4], with some data even projecting that the growth in demand for shoulder arthroplasty might exceed that of hip and knee arthroplasty in the future [5]. A nation-wide registry study in Finland showed a 160% increase in primary shoulder arthroplasty during 2004–2015 [3]. Similar data were reported in another study in Italy showing a three-fold increase in this procedure between 2009 and 2019 and a projected 72.3% increase in the next ten years [4].

Considering the essential role shoulder arthroplasty plays in addressing a spectrum of glenohumeral and rotator cuff conditions, it is crucial to identify elements that could influence long-term efficacy of this procedure. Different studies have shown that bone health and integrity have an impact on the outcome of prosthetic surgery [6,7,8,9]. Complications such as periprosthetic fracture, aseptic loosening or infection may arise as a consequence of compromised bone integrity and bone loss. Most of these studies focus on either hip or knee arthroplasty. Moreover, there are currently even less data available on the role of bone quality comparing lower extremity arthroplasty to upper extremity arthroplasty. The major difference between these two is that hips and knees are weight-bearing joints. It is well known that weight-bearing is associated with bone ingrowth in arthroplasty. As this does not apply for upper extremity arthroplasty, the necessity for a healthy bone metabolism might be even higher in these patients.

An essential element for optimal bone health and integrity is vitamin D. Vitamin D plays a crucial role in maintaining bone health by regulating calcium and phosphate homeostasis, thereby ensuring a balanced bone metabolism, characterised by an equilibrium between bone resorption and formation [10,11]. Several studies have shown an association between inadequate serum vitamin D levels and osteomalacia, osteoporosis, and muscle-weakness, all of which are important in the field of orthopaedic surgery [12,13].

Vitamin D deficiency is a concerning global problem. It is estimated that one billion people worldwide are affected by vitamin D deficiency, while 50% of the global population have vitamin D insufficiency [14]. Inadequate serum vitamin D levels not only negatively affect bone metabolism but are also related to an increased risk of postoperative complications after TJA, such as periprosthetic infection or postoperative stiffness and the necessity of revision surgery [15,16]. In the past few years, there has been growing interest in prehabilitation, a process that aims to improve the functional capability of a patient prior to a surgical procedure. In prehabilitation programmes for arthroplasty, physical training is often combined with nutritional supplementation. This aims to optimise a patient’s physical and nutritional status before joint replacement surgery, thus potentially leading to improved surgical outcomes and faster recovery. Vitamin D supplementation might also be of importance in patients who have been screened and are deficient in vitamin D at the preoperative stage. However, there are currently very few studies investigating vitamin D levels in patients undergoing TJA. Moreover, the exact prevalence of vitamin D deficiency for this patient collective is still largely unknown. For this reason, the main objective of this study was to determine the prevalence of hypovitaminosis D in patients scheduled for total shoulder or elbow arthroplasty.

## 2. Patients and Methods

Between May 2023 and May 2024, the serum 25OHD levels of patients scheduled for either shoulder or elbow arthroplasty at the Orthopaedic Department, König Ludwig Haus, University of Wuerzburg, Germany (situated at 49.5 degrees north latitude), were measured on admission. Furthermore, the serum levels of parathyroid hormone (PTH) and calcium of each study participant were regularly assessed.

Blood samples were usually collected one day before surgery. Written informed consent was obtained from all patients prior to blood collection. Serum 25OHD and PTH measurements were evaluated in a standardised manner by the Elecsys Vitamin D total III Assay (Roche Diagnostics GmbH, Mannheim, Germany) and Elecsys PTH (1–84) assay (Roche Diagnostics GmbH, Mannheim, Germany) for cobas^®^ e411 Analyzer (Roche Diagnostics; Mannheim, Germany). The laboratory results were collected using a retrospective chart review.

Exclusion criteria for this study were as follows: (1) patients receiving shoulder or elbow arthroplasty due to fracture; (2) patients with a reported osteoporosis medication such as bisphosphonates, Teriparatide or Denosumab; and (3) patients under the age of 18 years.

Collectively, 108 patients (*n* = 105 for shoulder arthroplasty, *n* = 3 for elbow arthroplasty) were included in the study. For data evaluation, recommendations of the US Endocrine Society were used defining vitamin D deficiency as serum 25(OH)D levels below 20 ng/mL (50 nmol/L). 25OHD levels between 20 and 29 ng/mL (50–72.5 nmol/L) were considered insufficient and 25OHD levels of 30 ng/mL (75 nmol/L) or above sufficient [17]. In this context, it should be noted that there is an ongoing debate regarding the definition of vitamin D deficiency. The optimal level of vitamin D is not universally agreed upon and currently different recommendations from various expert groups exist.

Demographics, comorbidities, and perioperative data were retrospectively collected from electronic medical records. Assessed data included factors that could potentially affect bone health, in line with the guidelines set forth by the German Osteology Society (Dachverband Osteologie, DVO) such as BMI, age, specific medication (e.g., Proton pump inhibitors, Glucocorticoids), and other comorbidities like diabetes or COPD.

As most of the body’s vitamin D is produced in the skin in a UVB-dependent manner, we also assessed the month of surgery of each patient to estimate daily sun exposure. Hence, we divided the cohort into four groups, depending on the average monthly sunshine hours in Germany (Spring: March–May; Summer: June–August; Autumn: September–November; Winter: December–February).

Statistical analyses were performed using IBM SPSS statistics software (IBM Corp., 2021, Version 28.0, Armonk, NY, USA). Patients’ characteristics were summarised using either means and standard deviations or frequencies and percentages. Distribution differences in categorical variables were assessed using the chi square test. Mean values of ordinal and interval-scaled variables were compared using parametric and non-parametric testing (student t, ANOVA). Relationships between variables were determined using regression analyses. *p*-values < 0.05 were considered statistically significant.

## 3. Results

A total of 108 patients undergoing either shoulder- or elbow arthroplasty were enrolled in this study. Of these, 31 patients (28.7%) reported regular vitamin D supplementation while 77 patients (71.3%) did not report regular vitamin D intake. Obesity (35.1%), followed by diabetes (18.5%) were the most frequent comorbidities. Further, 43.5% of patients regularly took medications that have previously been identified as a potential risk factors for osteoporosis. The most frequently described medications were protone pump inhibitors (35.2%) (Table 1).

**Figure 1:**
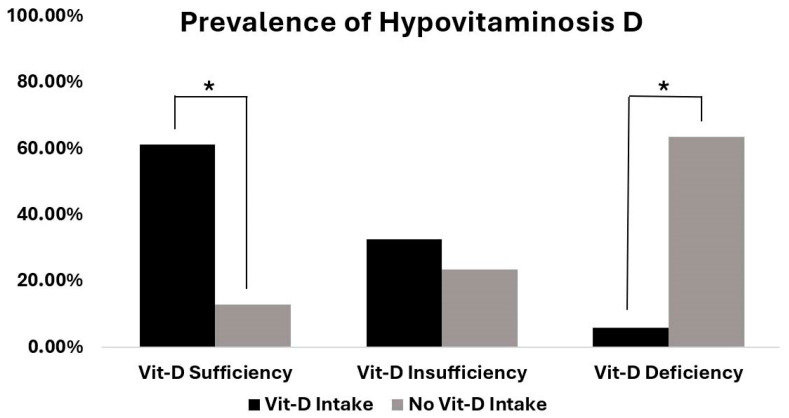
Prevalence of vitamin D sufficiency, insufficiency, and deficiency in patients with and without regular vitamin D intake. Asterisks mark significant differences between the two groups (*p* < 0.001).

A total of 77 patients (71.3%) scheduled for either shoulder or elbow arthroplasty did not regularly take any vitamin D supplements. Of these, 47 (61%) were women and 30 (39%) were men. The mean age was 70.2 ± 10.7 years, with a range from 44 to 89 years. Collectively, 87% (67/77) of these patients had low vitamin D levels (<30 ng/mL). Notably, 63.6% (49/77) of patients were vitamin D-deficient (<20 ng/mL), while only 13% (10/77) of these patients were within the target range of 30 to 50 ng/mL (Figure 1).

Out of the 31 patients with a reported regular vitamin D intake, 22 (70.1%) were women and 9 (29.9%) were men. The mean age was 73 ± 10.8 years, with a range from 47 to 88 years. In this group of patients, 62.3% (19/31) had sufficient vitamin D levels. Consequently, 38.7% (12/31) had inadequate vitamin D levels. Of these, 6% (2/31) had vitamin D levels <20 ng/mL (Figure 1).

**Figure 2:**
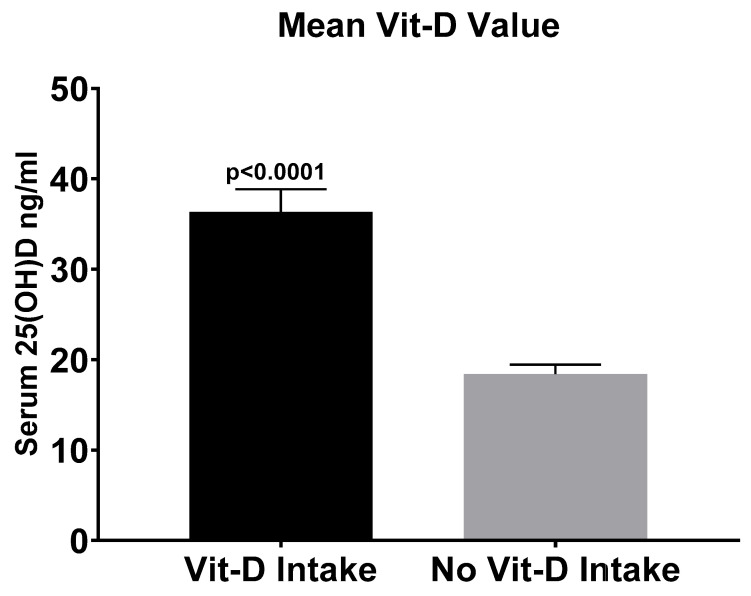
Mean serum 25(OH)D level of patients with and without vitamin D substitution (mean 36.4 ng/mL; *n* = 31 for vitamin D substitution and mean 18.4 ng/mL; *n* = 77 for no vitamin D substitution). There were significant differences between the two groups (*p* < 0.0001). Results are shown as means ± SEM.

Mean serum vitamin D level of all 77 patients without vitamin D supplementation was 18.4 ± 9 ng/mL. In contrast, mean serum vitamin D level of patients with a regular vitamin D intake was 36.4 ± 14 ng/mL (*p* < 0.0001) (Figure 2).

Additionally, a potential correlation of preoperative vitamin D levels and the use of cemented vs. non-cemented implants was evaluated. However, mean serum vitamin D levels did not differ significantly between cemented and non-cemented implants (cemented implants: *n* = 30, 18.7 ± 7.8 ng/mL; non-cemented implants: *n* = 45, 18.3 ± 9.9 ng/mL, *p* = 0.4).

**Figure 3:**
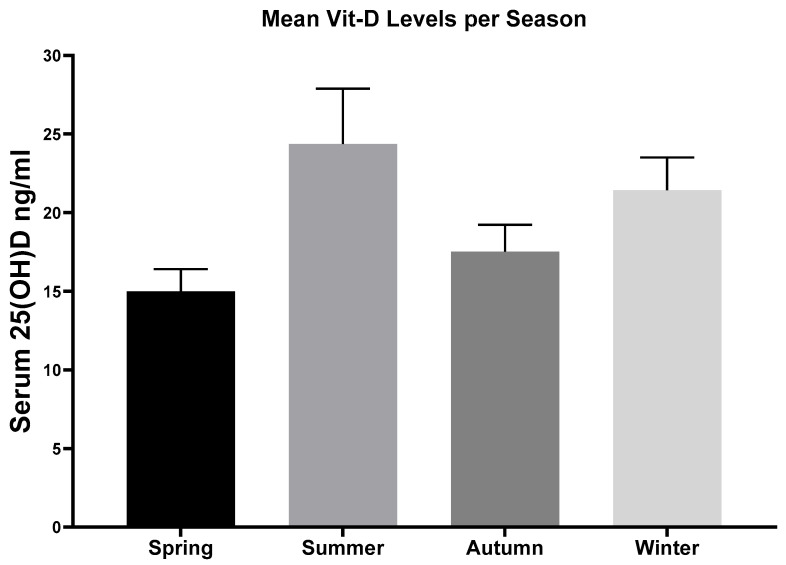
Seasonal variation in mean vitamin D levels. The lowest serum 25OHD levels were observed in spring (15 ± 7.3 ng/mL), followed by autumn (17.5 ± 8.2 ng/mL). The highest serum 25OHD levels were observed in summer (24.4 ± 11.7 ng/mL). Statistical analysis showed significant differences between spring and summer (*p* = 0.005). Results are shown as mean ± SEM.

Furthermore, mean serum vitamin D levels differed significantly with respect to seasonal variation (*p* = 0.01). Mean serum vitamin D level were lowest in spring (15 ± 7.3 ng/mL) and highest in summer (24.4 ± 11.7 ng/mL) (*p* = 0.005) (Figure 3).

A total of 11 patients (14.2%) with no reported vitamin D intake had elevated levels of parathyroid hormone (PTH). All of these exhibited secondary hyperparathyroidism as a result of severe vitamin D deficiency. Two of these patients also had low serum calcium levels (see Supplementary Table S1).

Regarding the analysis of potential risks factors for hypovitaminosis D, we identified that patients with a BMI > 30 kg/m^2^, without any regular vitamin D intake, had especially low 25OHD levels (*n* = 27, mean 17.5 ± 9.1 ng/mL). Compared to patients with a BMI > 30 kg/m^2^ and regular vitamin D intake (*n* = 19, 32.2 ± 11.3 ng/mL), significant differences were found (*p* < 0.01).

Furthermore, study participants older than 70 years of age and regular vitamin D supplementation had significantly higher mean serum vitamin D level (*n* = 21, 38.4 ± 13.4 ng/mL) compared to patients above the age of 70 and no regular vitamin D intake (*n* = 42, 17.1 ± 9.1 ng/mL) (*p* < 0.001). Comparing females to males, we did not find any significant differences in mean vitamin D levels (*n* = 39, 21.6 ± 11 ng/mL for men and *n* = 69, 24.7 ± 14.5 ng/mL for women) (*p* = 0.1). Likewise, no significant difference was observed between smokers (*n* = 18, 19.8 ± 9.3 ng/mL) and non-smokers (*n* = 86, 23.9 ± 13.6 ng/mL) (*p* = 0.1).

A multiple linear regression analysis was conducted to assess the impact of sex, age, BMI, and nicotine abuse as potential predictive variables for preoperative vitamin D serum levels. The analysis demonstrated that none of these variables were significant predictors of preoperative low vitamin D serum levels (Table 2 and Table 3).

## 4. Discussion

The main finding of this study is a high prevalence of hypovitaminosis D in patients undergoing shoulder or elbow arthroplasty. Furthermore, we observed a seasonal variation of 25(OHD) plasma levels, with the lowest levels registered in spring season.

Numerous studies have recently portrayed a high prevalence of vitamin D insufficiency in orthopaedic patients in general [18,19]. Similarly to our findings, high rates of vitamin D deficiency have also been reported in patients undergoing total joint arthroplasty (TJA) of the shoulder, hip, or knee joint [20,21,22]. For example, a single-centre analysis by Piuzzi et al. demonstrated a high prevalence of vitamin D insufficiency (60.6%) and deficiency (26.9%) in patients scheduled for primary joint arthroplasty of the lower extremity [21]. However, there is currently a paucity of data on the prevalence of hypovitaminosis D in patients undergoing arthroplasty of the upper extremity. In a retrospective study by Inkrott and colleagues, it was reported that nearly half of the study participants undergoing shoulder arthroplasty (43%) were vitamin D-insufficient, while further 11% of the patients were vitamin D-deficient [22]. Another recent retrospective study from McConnell et al. found that 29.9% of patients undergoing shoulder arthroplasty had insufficient vitamin D levels and further 35.11% had deficient levels of vitamin D [23]. Our present study revealed an even higher prevalence of vitamin D insufficiency (87%) in this patient collective. To the best of our knowledge, this is the first study reporting on the prevalence of hypovitaminosis D in patients undergoing TJA of the upper extremity in a relatively large cohort in Germany.

Vitamin D plays an important role in the musculoskeletal system [10]. In addition to its role in maintaining bone health by regulating bone metabolism, there is clear evidence that vitamin D is also crucial for optimising muscle strength and performance [10,24]. Hence, inadequate levels of vitamin D in patients undergoing TJA have been shown to affect postoperative patient outcomes [9,16]. A meta-analysis of Vivek et al. revealed that vitamin D deficiency was associated with poorer functional outcome scores, as well as an elevated risk of revision surgery, an increased level of periprosthetic joint infection, and postoperative stiffness in patients following total knee arthroplasty [16]. Smith and colleagues reported that vitamin D deficiency is associated with a higher rate of all-cause revision total shoulder arthroplasty [9]. Furthermore, a number of studies have indicated an inverse correlation between low vitamin D levels and the length of hospitalisation of patients undergoing elective TJA [16,25].

Consequently, vitamin D supplementation has been shown to improve postoperative outcome of TJA patients [16,26,27]. For example, a nationwide population-based cohort study of Kong and colleagues reported that the combination of calcium and vitamin D with a dose of 800 IU or greater (for more than 1 year) was associated with the most significant reduction in the risks for revision surgery after total knee arthroplasty [27]. A study from Maniar et al. reported that patients with inadequate serum vitamin D levels undergoing total knee arthroplasty could be rapidly supplemented in the morning of surgery with a large dose of oral cholecalciferol 600,000 IU, and the effect was consistent over 2 weeks after surgery [28]. In contrast, a randomised controlled trial from Weintraub et al. failed to demonstrate an improvement in functional- and patient-reported outcomes following the administration of 50,000 international units of vitamin D_3_ on the day of surgery for patients undergoing TKA [29]. Therefore, further randomised controlled trials are needed to evaluate if there is a causal relationship between low vitamin D levels and poor patient outcomes.

Given the emerging evidence that low vitamin D levels potentially lead to poorer postoperative outcomes, it is noteworthy that only 28.7% of the participants in our present study reported a regular vitamin D intake. This proportion remains low despite potential awareness. It should also be noted that only 13.0% of the patients without a vitamin D intake had sufficient 25OHD levels, although low vitamin D levels in patients scheduled for TJA had previously been associated with poor outcomes. In line with this, the prevalence of vitamin D deficiency was also found to be high in patients that require revision arthroplasty [30]. In a separate study, Smith and colleagues found that only 28.3% of patients undergoing shoulder arthroplasty reported taking vitamin D supplements [9]. Importantly, the majority of patients in our study that reported a regular vitamin D intake had sufficient vitamin D levels. This suggests that vitamin D supplementation was effective in our cohort.

An analysis for risk factors of vitamin D deficiency did not show any significant correlation between vitamin D levels and BMI. This was somewhat surprising because several studies have shown an inverse association between vitamin D plasma levels and body mass index (BMI) [31,32]. Further, sex, age, or smoking status also failed to be independent risks factors for low vitamin D levels. In contrast to our findings, a meta-analysis from Yang et al. revealed that the level of circulating 25OHD in smokers was lower than that in nonsmokers [33].

The current study also demonstrates a seasonal variation in mean vitamin D levels, with the lowest mean vitamin D level registered in spring and the highest in summer. In accordance with our findings, numerous studies have similarly documented a seasonal variation in vitamin D levels, with the highest levels observed in summer and the lowest levels occurring in spring and winter [21,34].

As with any single-centre analysis, this retrospective study is not without limitations. For instance, due to the chart review method we do not have any precise data on the type or dosage of vitamin D supplementation of each patient with a reported intake of vitamin D, nor do we know the daily sunlight exposure of the study participants. These results therefore need to be interpreted with caution. Further research is required to evaluate the impact of different types of vitamin D supplementation and dosing recommendations before drawing any conclusions. Moreover, the geographical location of Wuerzburg (49°5′ N) limits our results to regions situated at approximately the same latitude (e.g., Paris, Vancouver, or Kiev).

In summary, this observational study with a relatively large number of patients provides valuable data on the prevalence of vitamin D deficiency in patients undergoing TJA of the upper extremity. Our data clearly show that patients scheduled for upper extremity TJA have an overall high prevalence of vitamin D insufficiency (87%). Notably, patients with a BMI > 30 kg/m^2^ and patients over the age of 70 years without any regular vitamin D intake have increased risk for hypovitaminosis D. Therefore, it might be advisable to regularly assess preoperative vitamin D levels, as part of the standard preoperative laboratory tests, in these patients. Moreover, it is likely that patients that were identified with inadequate vitamin D levels would benefit from vitamin D substitution. However, this study is limited by the absence of precise information on the type and dosing of vitamin D supplementation. Further, this study is limited by the fact that it only investigates the prevalence of vitamin D deficiency. Data on the postoperative outcomes of patients, which could provide more definitive evidence on a positive effect of vitamin D supplementation, is lacking. For this reason, further studies need to be carried out in order to validate the potential effect of vitamin D supplementation in these patients.

## 5. Conclusions

In the present study, we found a high rate of hypovitaminosis D (87% of patients without regular vitamin D intake) among orthopaedic patients scheduled to undergo elective shoulder or elbow arthroplasty. For this reason, the assessment of serum vitamin D levels for this patient collective as part of the standard preoperative laboratory tests is recommended.

## Figures and Tables

**Table 1:** Patient characteristics.

Characteristics	Values (Mean, Value, Precent)
No. of patients	108
**Sex**	
Men	39 (36.6%)
Women	69 (63.4%)
**Mean Age (years ± SD)**	71 ± 10.7
**Reason for admission**	
Shoulder arthroplasty	105 (97.2%)
Elbow arthroplasty	3 (2.8%)
**Comorbidities**	
Nicotine abuse	18 (16.7%)
Obesity (BMI ≥ 30 kg/m^2^)	27 (25.0%)
Tumour disease	4 (04.0%)
Rheumatoid arthritis	4 (4.0%)
Diabetes	20 (18.5%)
Pulmonary disease (COPD)	5 (5.0%)
**Medication**	
Glucocorticoids	12 (11.1%)
Protone pump inhibitors (PPI)	38 (35.2%)
Vitamin D supplementation, oral	31 (28.7%)

BMI: body mass index; COPD: chronic obstructive pulmonary disease.

**Table 2:** Analysis of risk factors for hypovitaminosis D.

Predictor	Coefficient	Std. Error	*p*-Value	Exp(B)	95% Confidence Interval for Exp(B)
Sex	−0.517	0.771	0.502	0.596	[0.132–2.693]
Age	−0.006	0.037	0.876	0.994	[0.924–1.069]
BMI	−0.051	0.070	0.463	0.950	[0.829–1.089]
Smoking	−0.965	1.233	0.434	0.381	[0.035–4.100]

**Table 3:** Logistic regression: predictors of vitamin D deficiency (<20 ng/mL). This table presents the adjusted odds ratios (OR) and 95% confidence intervals for predictors of vitamin D deficiency (<20 ng/mL), including age, BMI, sex, season, and vitamin D supplementation.

Predictor	OR	95% CI	*p*-Value
Sex [Male]	1.371	0.485–3.88	0.552
Season [Autumn]	2.094	0.47–9.33	0.333
**Season [Spring]**	4.323	1.037–18.013	**0.044**
Season [Winter]	0.648	0.125–3.36	0.606
Age	1.006	0.963–1.05	0.793
BMI	1.079	0.987–1.18	0.095
**Supplement**	0.062	0.013–0.305	**0.001**

## Data Availability

Data available on request due to restrictions (privacy/ethical reasons).

## Supplementary Materials

The following supporting information can be downloaded at: https://www.mdpi.com/article/10.3390/nu17162635/s1. Table S1: 25(OH)D-, PTH- and Calcium Levels in Patients with and without Vitamin D Supplementation, Across Subgroups (mean ± SD).
