# Supplementary material for: High Prevalence of Vitamin D Deficiency in Patients Undergoing Total Shoulder or Elbow Arthroplasty
Source: Nutrients. 2025 Aug 14;17(16):2635. doi: 10.3390/nu17162635 (PMC12389540; doi:10.3390/nu17162635)
Supplement: Supplementary file 1 [file nutrients-17-02635-s001.zip › nutrients-3769392-supplementary.pdf]

Supplementary Data

Supplementary Table S1. 25(OH)D-, PTH- and Calcium levels in patients with and without vitamin D supplementation across subgroups (mean ± SD)

Subgroup	N (without)	25(OH)D (ng/ml)	PTH (ng/l)	Ca (mmol/l)	N (with)	25(OH)D (ng/ml)	PTH (ng/l)	Ca (mmol/l)
**Sex**								
Female	47	18.77 (±9.60)	48.14 (±19.78)	2.41 (±0.14)	22	37.36 (±15.21)	69.66 (±60.12)	2.42 (±0.11)
Male	30	17.90 (±8.07)	52.51 (±20.83)	2.43 (±0.13)	9	33.89 (±10.91)	46.00 (±27.00)	2.44 (±0.12)
**Age (years)**								
< 70	43	20.06 (±8.76)	42.55 (±16.30)	2.47 (±0.10)	10	32.00 (±14.94)	48.19 (±27.84)	2.51 (±0.12)
≥ 70	34	17.14 (±9.07)	55.69 (±21.22)	2.37 (±0.15)	21	38.43 (±13.41)	69.75 (±61.31)	2.40 (±0.10)
**BMI (kg/m^2)**								
< 30	43	19.84 (±9.29)	50.98 (±21.55)	2.41 (±0.13)	20	37.10 (±14.23)	60.75 (±63.81)	2.43 (±0.11)
30–39.9	21	18.05 (±9.43)	43.94 (±13.17)	2.41 (±0.17)	10	32.20 (±11.28)	69.21 (±27.66)	2.45 (±0.14)
≥ 40	6	15.50 (±8.57)	55.50 (±23.75)	2.41 (±0.12)	1	63.00	39.50	2.42
**Smoking**								
Yes	14	16.57 (±6.82)	40.43 (±18.18)	2.48 (±0.11)	4	31.25 (±8.18)	30.48 (±5.59)	2.50 (±0.04)
No	60	18.60 (±9.37)	52.33 (±20.51)	2.40 (±0.14)	26	36.12 (±13.96)	68.66 (±56.42)	2.43 (±0.12)
